# Altitude Travel in Patients With Pulmonary Hypertension: Randomized Pilot-Trial Evaluating Nocturnal Oxygen Therapy

**DOI:** 10.3389/fmed.2020.00502

**Published:** 2020-09-02

**Authors:** Mona Lichtblau, Stéphanie Saxer, Tsogyal D. Latshang, Sayaka S. Aeschbacher, Fabienne Huber, Philipp M. Scheiwiller, Joël J. Herzig, Simon R. Schneider, Elisabeth D. Hasler, Michael Furian, Konrad E. Bloch, Silvia Ulrich

**Affiliations:** Department of Pulmonology, University Hospital Zurich, Zurich, Switzerland

**Keywords:** pulmonary hypertension, altitude, oxygen, exercise performance, sleep, echocardiography

## Abstract

**Introduction:** Stable patients with pulmonary arterial or chronic thromboembolic pulmonary hypertension (PH) wish to undergo altitude sojourns or air travel but fear disease worsening. This pilot study investigates health effects of altitude sojourns and potential benefits of nocturnal oxygen therapy (NOT) in PH patients.

**Methods:** Nine stable PH patients, age 65 (47; 71) years, 5 women, in NYHA class II, on optimized medication, were investigated at 490 m and during two sojourns of 2 days/nights at 2,048 m, once using NOT, once placebo (ambient air), 3 L/min per nasal cannula, according to a randomized crossover design with 2 weeks washout at <800 m. Assessments included safety, nocturnal pulse oximetry (SpO_2_), 6-min walk distance (6 MWD), and echocardiography.

**Results:** At 2,048 m, two of nine patients required medical intervention, one for exercise-induced syncope, one for excessive nocturnal hypoxemia (SpO_2_ < 75% for >30 min). Both recovered immediately with oxygen therapy. Two patients suffered from acute mountain sickness. In 6 patients with complete data, nocturnal mean SpO_2_ and cyclic SpO_2_ dips reflecting sleep apnea significantly differed from 490 to 2,048 m with placebo, and 2,048 m with NOT (medians, quartiles): SpO_2_ 93 (91; 95)%, 89 (85; 90)%, 97 (95; 97)%; SpO_2_ dips 10.4/h (3.1; 26.9), 34.0/h (5.3; 81.3), 0.3/h (0.1; 2.3). 6 MWD at 490, 2,048 m without and with NOT was 620 m (563; 720), 583 m (467; 696), and 561 m (501; 688). Echocardiographic indices of heart function and PH were unchanged at 2,048 m with/without NOT vs. 490 m.

**Conclusions:** 7/9 PH patients stayed safely at 2,048 m but revealed hypoxemia, sleep apnea, and reduced 6 MWD. Hemodynamic changes were trivial. NOT improved oxygenation and sleep apnea. The current pilot trial is important for designing further studies on altitude tolerance of PH patients.

## Introduction

Precapillary pulmonary hypertension (PH) is defined as mean pulmonary artery pressure (PAP) > 20 mmHg and pulmonary artery wedge pressure (PAWP) ≤ 15 mmHg along with a pulmonary vascular resistance (PVR) ≥ 3 WU assessed by right heart catheterization (1). Since 1998, PH has been classified into five major groups according to clinical presentation and response to vasodilator and antiproliferative therapies (1). In the absence of predominant lung or certain rare diseases, the major precapillary PH forms are pulmonary arterial hypertension (PAH) and chronic thromboembolic PH (CTEPH), defined as groups I and IV and below summarized as PH and these groups are also summarized as pulmonary vascular diseases (PVD) (1). The leading symptom in PH is dyspnea on exertion with impaired exercise performance and restrained daily activity (2). With progression of the disease, worsening hemodynamics may lead to gas exchange disturbances associated with hypoxemia, particularly during exercise and sleep (3, 4). Current guidelines recommend that PH patients with hypoxemia or exertional dyspnea or in New York Heart Association functional class ≥III avoid traveling to altitudes above >1,500 m and use supplemental oxygen during air travel (5). However, these recommendations are not based on scientific evidence. In a recent review of the literature, no conclusive studies regarding the effect of a hypoxic environment at altitude on patients with preexisting PH was found (6). A nonrandomized small study in patients with PAH exposed for 20 min to normobaric hypoxia with an inspiratory oxygen fraction (FiO_2_) of 15% corresponding to an altitude of ≈2,500 m revealed that resting PAP assessed by echocardiography did not increase (7). Moreover, we recently showed that exposure to normobaric hypoxia (FiO_2_ 15%) during right heart catheterization did not significantly change invasive pulmonary hemodynamics at rest in patients with PAH/CTEPH despite a decrease in arterial oxygen tension from 9.5 to 7.0 kPa (8). It is not known whether and to what extent environmental hypoxia during hours to days as in air travel or altitude sojourns is harmful for patients with PH, and potential risk factors are not identified. In our previous short-duration study with normobaric hypoxia, PH patients revealed an even smaller fall in arterial oxygen tension compared to unaffected controls while breathing hypoxia during 15′, related to increased hyperventilation as indicated by the significant decrease in arterial partial pressure of carbon dioxide (PaCO_2_) (8). In healthy volunteers, exercise performance is gradually impaired with increasing hypoxia/altitude (9) and even more in patients with COPD (10, 11) and improves with adaptation (12). Data on PH patients exercising at altitude is completely lacking.

Worldwide, millions of people travel regularly to higher settlements or mountain areas for business or recreational purposes and expose themselves to hypobaric hypoxia during days to weeks or even longer. In addition, traveling by airplane, which has become extremely popular, further increased the number of people that are transiently exposed to a hypoxic environment, as the lower limit of cabin pressure is equivalent to 2,430 m (8,000 ft) of altitude. Very little is known about the cardiovascular adaptive changes and risks during exposure to hypoxia in PH patients. In our daily practice, many PH patients ask whether they can travel with their relatives and friends to the nearby Alps or undergo air travel for business or recreational purposes.

Therefore, the purpose of the current pilot trial in PH patients was to evaluate safety, echocardiographic indices of cardiac function, exercise performance, and sleep-related breathing disturbances during a stay at moderately high altitude and to evaluate the hypothesis that nocturnal oxygen therapy (NOT) during the altitude sojourn would improve these outcomes.

## Materials and Methods

### Study Design

This randomized, placebo-controlled, blinded crossover pilot trial was performed from January to October 2014, in PH patients permanently living <800 m. The study was approved by the Cantonal Ethics Committee Zurich (EK-2013-0088), and all participants signed a written informed consent. The trial is registered at clinicaltrials.gov, NCT02150616.

It evaluated the effects of NOT vs. placebo (nocturnal ambient air) during two sojourns at 2,048 m on symptoms, safety, sleep disordered breathing (SDB), vital signs, exercise performance, and hemodynamics by echocardiography. Participants underwent baseline evaluations during daytime and an overnight stay at 490 m (Zurich, Switzerland) and had the same evaluations over the course of 2 sojourns of 2 days/nights each in a mountain hotel at 2,048 m (St. Moritz, Switzerland). During the nights at 2,048 m, patients received either NOT or placebo (3 l/min) according to a randomized crossover trial via a nasal cannula. To minimize carryover effects, patients had to spend at least 2 weeks at low altitude (<800 m) between the two altitude sojourns.

### Patients

Patients with pulmonary arterial or chronic thromboembolic PH living below 800 m in New York Heart Association functional class II–III were recruited at the University Hospital of Zurich.

Patients with unstable or exacerbated condition, severe PH (functional class IV), concomitant unstable cardiovascular disease, hypoventilation, severe hypoxemia needing long-term oxygen therapy at low altitude or drugs that affect respiratory center drive, previous intolerance of moderate altitude (<2,600 m), or exposure to altitudes >1,500 m for >2 days within the last 4 weeks before the study were excluded.

### Randomization

Patients were randomized to receive either NOT or nocturnal placebo (ambient air) at altitude. Participants traveled by train and car within 3 h from 490 m to 2,048 m. Between altitude sojourns, a >2-week washout period <800 m was imposed.

### Intervention

Patients received NOT or placebo (ambient air) during nights at 2,048 m provided via nasal prongs connected to a concentrator (EverFlo, Philips Respironics, Zofingen, Switzerland) with a constant flow rate of 3 l/min. Patients were blinded for the intervention by placing the concentrators in a separate room.

For safety reasons, SpO_2_ was permanently monitored by the investigators at night and patients received oxygen, when SpO_2_ was <75% for more than 30 min during the sojourn at 2,048 m altitude or in the presence of symptoms requiring an intervention.

### Assessments

All symptoms, sings, or events relevant for safety and/or requiring medical intervention were noted, in particular excessive hypoxemia (SpO_2_ <75% for > 30 min) and acute mountain sickness (AMS), among others.

AMS was evaluated with the Environmental Symptoms Questionnaire cerebral score (AMSc). AMSc scores ≥ 0.7 were considered to be clinically relevant AMS (13).

The 6-min walking test was performed according to guidelines (14). Arterial blood gases were obtained at low altitude and at high altitude after the 1^st^ night.

Patients performed an incremental (ramp) exercise test on arrival (before NOT resp. placebo) and a constant work-rate exercise test on day 2 during each sojourn (60% of the maximal work rate achieved in the incremental work-rate test at lowland).

Echocardiography was performed in Zurich and on day 2 (after the first night) at altitude according to previously published studies and included assessments of right ventricular function (right ventricular fractional area change (RV-FAC), tricuspid annular plane systolic excursion (TAPSE), and peak systolic right ventricular to right atrial pressure gradient (RV/RA gradient) (15). Echocardiographic recordings were performed with a real-time, phased array sector scanner (CX 50, Philips, Philips Respironics, Zofingen, Switzerland) with an integrated Color Doppler system and a transducer containing crystal sets for imaging (1–5 MHz) and for continuous-wave Doppler. Measurements were carried out according to guidelines of the European Association of Echocardiography (16).

Patients had a respiratory sleep study (Alice 5, Philips Respironics) according to international standards (17, 18) at low and high altitude. Apneas/hypopneas were scored when there was a reduction of nasal pressure swings or the inductive plethysmographic sum signal to <50% of baseline for >10 s, as described previously (19–21) (Transient reductions in breathing amplitude to <50% of baseline for 5–10 s were also scored as apneas/hypopneas if they occurred as part of a periodic breathing pattern with hyperventilation alternating with central apneas/hypopneas for at least three consecutive cycles.). The apnea/hypopnea index (AHI) was computed as the number of events per hour of total sleep time and time in bed; the oxygen desaturation index (ODI, > 3% SpO_2_ dips) was computed as the number of events per hour of time in bed as described previously (22).

### Outcomes

Main endpoints were safety and tolerability of altitude exposure, blood oxygenation (SpO_2_ and blood gases), exercise performance (6-min walking distance (6 MWD), cycle endurance time), hemodynamics/heart function by echocardiography, sleep-related breathing disturbances, and the effect of NOT on these parameters.

### Statistics

In this pilot study, measurements are presented for each patient separately and/or summarized for the six patients who were treated by protocol as median and quartiles. Comparisons between summarized measures were performed with the Wilcoxon test. *P* < 0.05 was considered as a statistically significant difference; all analyses were performed with the statistical program SPSS 25 (SPSS, Chicago, IL, USA).

## Results

**Figure 1:**
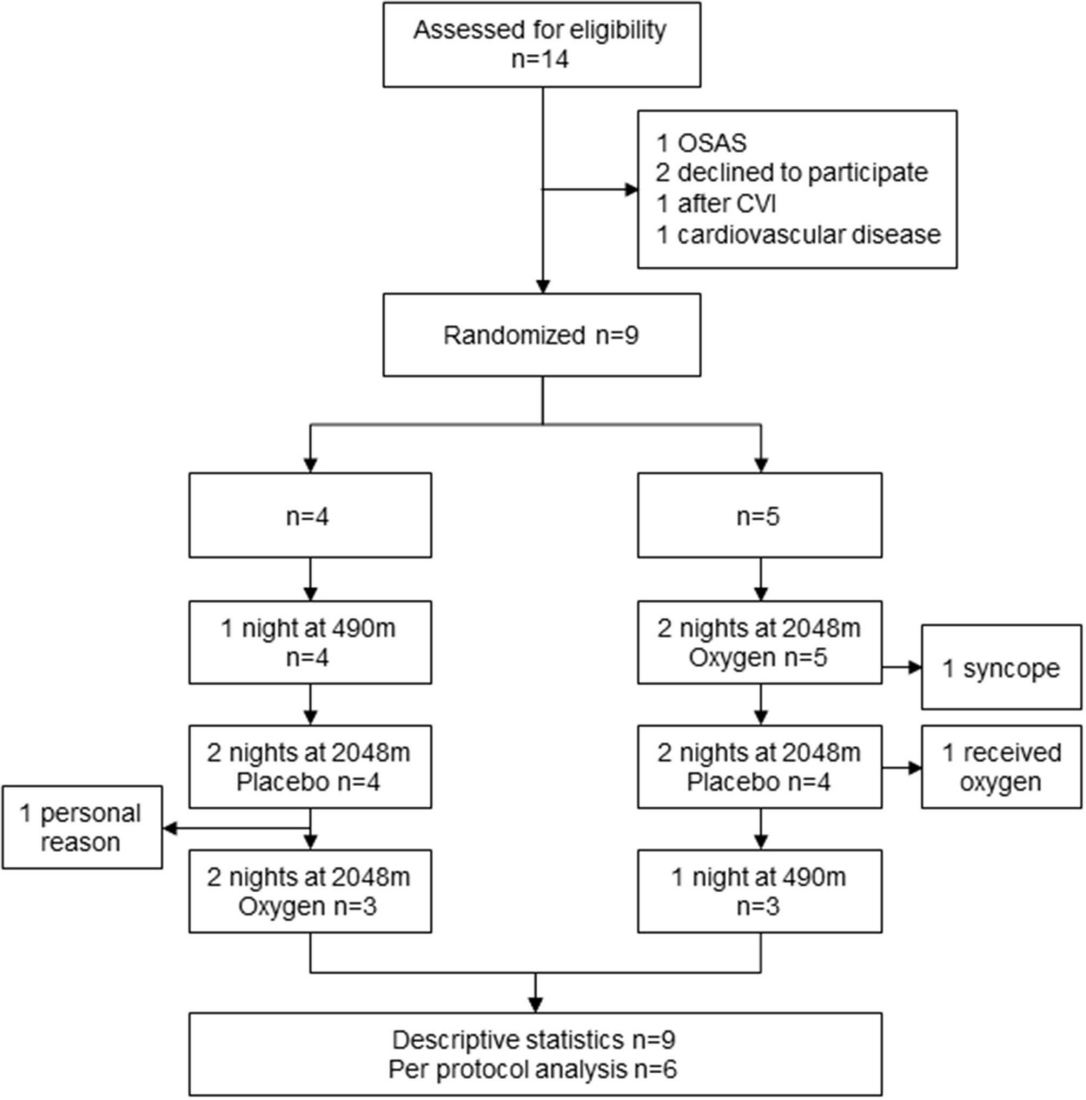
Patient flow. OSAS, obstructive sleep apnea syndrome; CVI, cerebrovascular insult.

Nine patients with PH were included in the study with a median age of 65 (49; 71) years, 5 women, and 5 CTEPH/4 PAH, all in NYHA functional class II; the study flow is presented in Figure 1. Baseline characteristics of the six patients who completed the study are shown in Table 1.

**Table 1:** Baseline characteristics given as median (IQR) or number (%).

***N***	**6**
Sex, male/female	3/3
Age	65 (49; 71)
Body mass index, kg/m^2^	24.4 (23.7; 26.8)
New York Heart Association functional class II	6 (100)
6-minute walking distance, m	620 (562; 720)
**Pulmonary hypertension group**	
Pulmonary arterial hypertension	2 (33)
Chronic thromboembolic pulmonary hypertension	4 (67)
**Right heart catheter**	
Mean pulmonary artery pressure, mmHg	36 (30; 43)
Pulmonary artery wedge pressure, mmHg	10 (9–10)
Pulmonary vascular resistance, WU	5.9 (5.2; 9.0)
Cardiac index, L·min^−1^·m^−2^	2.2 (2.0; 2.4)
**Pulmonary hypertension treatment**	
Endothelin receptor antagonist	3 (50)
Phosphodiesterase-5 inhibitors	2 (33)
Combination therapy	1 (17)
Calcium channel blockers	1 (17)

*PaO_2_, arterial partial pressure of oxygen; PaCO_2_, arterial partial pressure of carbon dioxide*.

One patient fulfilled the predefined safety criterion of SpO_2_ <75% for more than 30 min during the sojourn at 2,048 m during the placebo phase and was treated with oxygen accordingly (Patient ID 2). Two other patients performed only one visit at altitude and therefore did not complete the protocol: one was due to personal reasons (Patient ID 4), and the other patient had a syncope during cardiopulmonary exercise testing the day of arrival (Patient ID 9). This patient and the other one suffered from AMS (Patient ID 9 and 6). Six patients completed the study procedure according to the protocol.

The arterial partial pressure of oxygen (PaO_2_) assessed by arterial blood gases was significantly lower only after the 1^st^ night with NOT compared to the low altitude baseline, but not compared to placebo, and PaO_2_ after placebo was similar to low altitude measures. PaCO_2_ did not significantly differ in all circumstances (see Table 2). Heart rate and systolic blood pressure at the end of 6 MWD were significantly higher at altitude, irrespective of the intervention (see Table 2).

**Table 2:** Vital signs, exercise tests, and blood gas analysis.

	**Baseline Zurich 490 m *n* = 6**	**St. Moritz 2048 m Placebo** ***n*** **=** **6**		**St. Moritz 2048 m NOT** ***n*** **=** **6**	
		**Day 1**	**Day 2**	**Day 1**	**Day 2**
**Vital signs**					
HR rest, 1/min	64 (56; 78)	66 (61; 77)	71 (65; 76)	70 (64; 72)	70 (62; 78)
BP rest, systolic, mmHg	114 (102; 142)	127 (121; 135)	127 (121; 135)	125 (117; 132)	121 (113; 141)
BP rest, diastolic, mmHg	74 (71; 89)	80 (72; 89)	80 (72; 88)	78 (71; 82)	76 (69; 89)
SpO_2_ pre 6 MWD, %	97 (94; 98)	95 (88; 96)^*^	95 (92; 95)	95 (90; 97)	95 (90; 95)
**6-minute walk test**					
6 MWD, m	620 (563; 720)	583 (467; 696)^*^	563 (485; 653)^*^	561 (501; 688)^*^	572 (500; 691)^*^
HR end 6 MWD, 1/min	94 (80; 106)	104 (95; 123)^*^	103 (84; 116)	100 (95; 116)^*^	108 (97; 149)^*^
BP end 6 MWD, systolic, mmHg	150 (146; 155)	175 (171; 186)^*^	177 (170; 183)^*^	175 (170; 191)^*^	173 (153; 197)
BP end 6 MWD, diastolic, mmHg	86 (77; 96)	93 (85; 100)	89 (79; 97)	83 (72; 119)	84 (72; 95)
SpO_2_ end 6 MWD, %	87 (82; 92)	83 (76; 87)^*^	80 (73; 87)	78 (69; 83)^#^	80 (75; 87)
Borg dyspnea	3.5 (1.6; 5.0)	4.5 (3.0; 7.2)	5.0 (3.0; 5.8)^*^	4.5 (3.0; 6.0)	4.5 (3.0; 7.8)
Borg fatigue	2.5 (0.8; 4.5)	5.0 (2.3; 7.0)^*^	5.0 (2.3; 5.3)	3.0 (2.3; 4.8)	4.0 (2.3; 6.0)
**Constant work-rate endurance test (CWRET) at 60% of Wmax**					
Endurance time, s	826 (243; 1410)	200(160; 785)^*^		424 (163; 694)	
PaO_2_ end CWRET, mmHg	59.7 (51.6; 67.4)	53.9 (43.9; 63.9)		54.6 (42.2; 60.7)	
SaO_2_ end CWRET, %	90.8 (87.1; 94.1)	83.3 (75.2; 91.7)		89.5 (75.7; 91.4)	
**Arterial blood gas analysis**					
pH	7.48 (7.42; 7.48)	7.46 (7.44; 7.49)		7.46 (7.48; 7.49)	
PaO_2_, mmHg	65 (60; 72)	60 (54; 63)		58(50; 61)^*^	
PaCO_2_, mmHg	32 (29–35)	32 (29–34)		33 (29–36)	
**Sleep study**					
SpO_2_, %	93 (91; 95)	89 (85; 90)^*^	90 (89; 92)^*^	97 (95; 97)^*^ ^#^	97 (95; 97)^*^ ^#^
ODI, 1/h	10.4 (3.1; 26.9)	34.0 (5.3; 81.3)^*^	27.2 (6.9; 61.5)^*^	0.3 (0.1; 2.3)^*^ ^#^	0.7 (0.1; 4.0)^*^ ^#^
AHI, 1/h	27.3 (17.5; 38.7)	34.2 (13.3; 71.2)	29.8 (16.5; 63.7)	23.9 (2.4; 30.7)	15.2 (7.7; 33.0)^*^ ^#^

*HR, heart rate; BP, blood pressure; 6 MWD, 6-minute walking distance; PaO_2_, arterial partial pressure of oxygen; PaCO_2_, arterial partial pressure of carbon dioxide; SpO_2_, oxygen saturation; ODI, oxygen desaturation index; AHI, apnea/hypopnea index*.

* *Significant difference to low altitude baseline p < 0.05*;

# *significant difference to placebo of the same night p < 0.05*.

**Figure 2:**
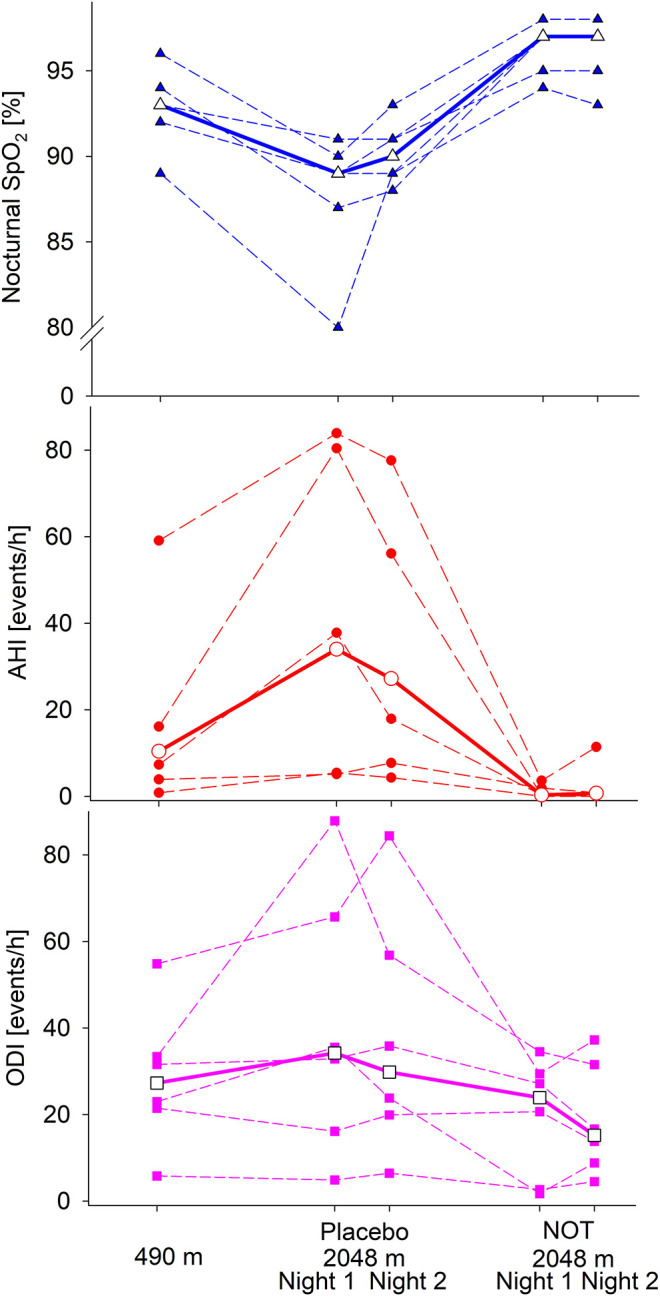
Six-minute walking distance (6 MWD) and oxygen saturation (SpO_2_) at the beginning and end of the 6 MWD of the patients who completed the study. Bold, median.

All patients had a reduced 6 MWD at 2,048 m compared to 490 m. Patients desaturated during 6 MWD in all phases. The lowest SpO_2_ was found at the end of 6 MWD at 2,048 m after receiving NOT, and between-group difference was only significant comparing desaturation at 490 vs. 2,048 m after having received NOT (*p* = 0.043), but not placebo, shown in Table 2 and Figure 2. Constant work-rate cycle endurance time was significantly reduced at altitude when patients received placebo, *p* = 0.046 (Table 2). All patients performed an incremental exercise test at lowland and shortly after arrival at altitude; however, the difference was not statistically significant (490 vs. 2,048 m (placebo): 109 (83; 201) vs. 103 (77; 187) W, p = ns).

Echocardiographic measures did not reveal significant differences between low and high altitude and between altitude sojourns with and without NOT (see Table 3).

**Table 3:** Echocardiographic measures of all patients listed separately and median (IQR) for *n* = 6 who performed the study according to protocol.

**ID**	**RV FAC (%) 490 m**	**RV FAC (%) 2,048 m Placebo**	**RV FAC (%) 2,048 m NOT**	**TAPSE (cm) 490 m**	**TAPSE (cm) 2,048 m Placebo**	**TAPSE (cm) 2,048 m NOT**	**RV/RA (mmHg) 490 m**	**RV/RA (mmHg) 2,048 m Placebo**	**RV/RA (mmHg) 2,048 m NOT**
1	25	21	24	2.2	2.7	2.4	53	46	43
3	43	29	42	2	1.4	1.7	22	24	25
5	37	44	47	2.8	2.5	2.7	10	21	16
6	39	43	30	2.7	2.4	2.19	53	77	68
7	32	48	40	2.2	2.3	3.4	17	19	31
8	41	37	38	2.3	2.2	2.5	26	22	38
Median (IQR)	38 (30–41)	40 (27; 46)	39 (29; 43)	2.3 (2.2; 2.7)	2.4 (2.0; 2.5)	2.4 (2.1; 2.9)	24 (15; 53)	24 (20; 54)	35 (22; 49)
2	30	34^†^	40	1.6	2.0^†^	2.2	28	40^†^	41
4	33	36		2.3	2.8		22	23	
9			33			1.8			86

*RV, right ventricle; FAC, fractional area change; TAPSE, tricuspid annular plane systolic excursion; RA, right atrium; NOT, nocturnal oxygen therapy*.

† *Patient received NOT in the placebo phase due to hypoxemia*.

**Figure 3:**
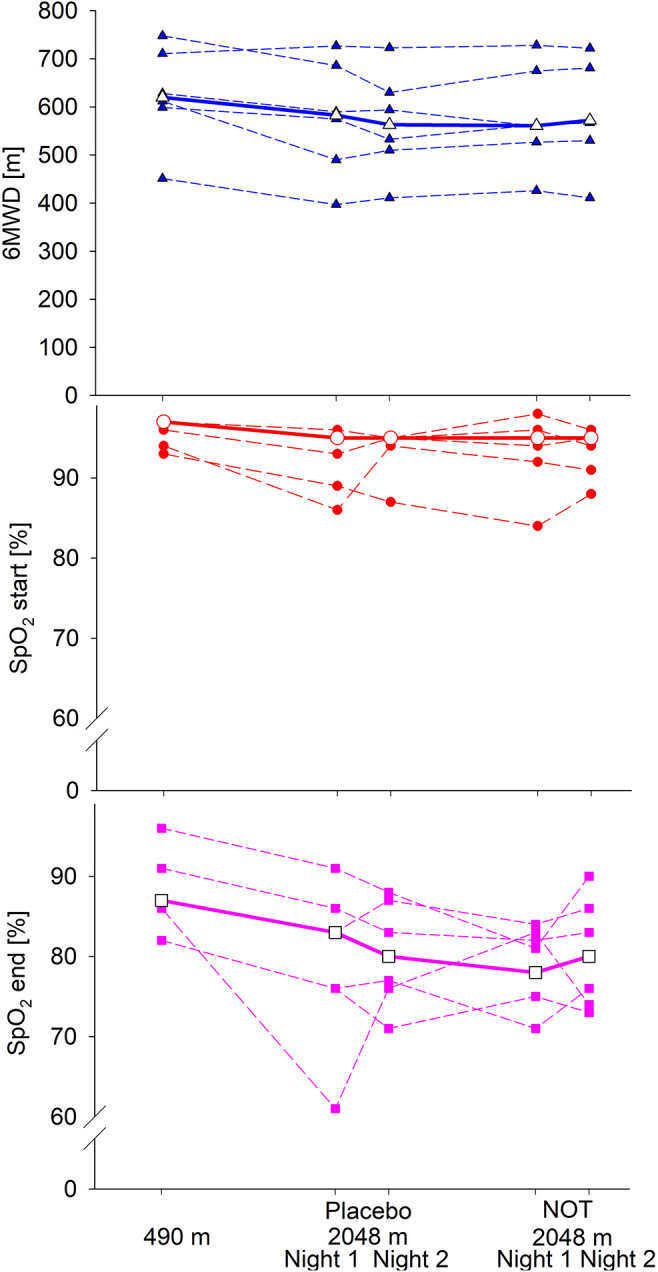
Sleep study results of the patients who completed the study are shown. SpO_2_, oxygen saturation; ODI, oxygen desaturation index; AHI, apnea/hypopnea index; Bold, median.

Altitude induced a significant decrease in nocturnal SpO_2_ and increase in ODI during both nights of the sojourn under placebo. AHI did not change significantly but tended to be higher at altitude. NOT at altitude significantly increased nocturnal SpO_2_ and decreased ODI and AHI compared to lowland and even more to altitude sojourns without NOT (Table 2 and Figure 3).

## Discussion

We performed a randomized, placebo-controlled, single-blind, crossover pilot trial to evaluate the effect of an altitude sojourn with and without NOT on safety and tolerability, blood oxygenation, exercise performance, hemodynamics, and SDB in patients with PH staying for 2 days and nights at moderate altitude (2,048 m). Exposure to altitude induced arterial hypoxemia and reduction of exercise performance, along with higher heart rates and blood pressures at end of exercise and increase in SDB. NOT improved SDB but not daytime exercise performance. Right heart function measured by echocardiography revealed no significant changes at altitude with or without NOT. Out of 9 patients, two had altitude-related adverse health effects, one patient needed oxygen, and another one had a syncope during cardiopulmonary exercise testing on the day of arrival. The latter patient and another one fulfilled criteria for AMS according to the AMSc questionnaire.

Clinical data and particularly randomized-controlled trials on patients with cardiorespiratory disorders, such as heart failure (HF) or COPD, traveling at altitude is scarce, and no data are available for patients with PH (6). In HF, altitude exposure is not recommended if patients are unstable (23) or suffer from comorbidities that may directly interfere with the adaptation to altitude (24). Only few studies are available in clinically stable patients with HF on optimal medical treatment investigating short exposure to real altitude or normobaric hypoxia simulating altitude near sea level (24, 25). One of those studies assessed exercise capacity in 29 optimally treated and stable patients with severe HF [ejection fraction <40%, peak oxygen uptake (peak VO_2_) > 50% predicted] traveling to 3,454 m (4–5-h stay at altitude) and revealed a reduction of 22.2% in the peak VO_2_ (26). Another study revealed no increase in the number of cardiac events in patients with coronary artery disease or HF with preserved ejection fraction exposed up to 3,500 m (23, 27, 28). Wyss et al. investigated patients with coronary artery disease under normobaric hypoxia (FiO_2_ 15%, equivalent to ~2,500 m) and found a decreased coronary flow reserve assessed by positron emission tomography on exercise (25).

Forty patients with moderate to severe COPD (median FEV_1_ 59% predicted) have been assessed in a randomized crossover trial ascending to 2,590 m, and a 24% incidence of altitude-related adverse health effects was reported, confirming the increased susceptibility in COPD patients exposed to moderate hypobaric hypoxia (29). In the same trial, left and right heart function was assessed by echocardiography, revealing an increase in pulmonary artery pressure and slight reduction in right ventricular function as well as an increase in diastolic dysfunction at altitude (15). At such altitudes, adverse events requiring medical treatment or descent have not been reported in healthy volunteers or in patients with obstructive sleep apnea syndrome (30, 31).

COPD guidelines recommend in-flight oxygen in patients with severe COPD when the hypoxic altitude simulation test reveals PaO_2_ <50 mmHg or SpO_2_ <85% (32). However, the role of this hypoxia-altitude-simulating test has never been established, as the degree of deoxygenation during the test hast not been shown to predict symptoms at real altitude (33). In line with this, a currently published trial has shown that 16% of moderate to severe COPD patients, who did not fulfill the criteria to perform a hypoxia-altitude simulation test, revealed severe deoxygenation at 2,048 m of real altitude (22).

Our study is the first to investigate lowlanders with stable but symptomatic PH during a 2-day/night altitude sojourn. We found that out of nine patients, two (22%) experienced altitude-related adverse health events, one severe nocturnal oxygen desaturation, the other exertional syncope, and two (22.2 %) fulfilled the diagnostic criteria of AMS, but these two did not need additional treatment. Despite the fact that altitude exposure was associated with a significantly lower blood oxygenation, pulmonary hemodynamics by echocardiography was not significantly different and also not altered with NOT at altitude. Similarly, no significant changes in right ventricular systolic function were found in our PH cohort. The patient number in this pilot study was too small to detect minor changes, but major hemodynamic decompensation was not observed. In a previous study in PH patients assessed during short-term exposure to normobaric hypoxia during right heart catheterization (15 min, FiO_2_ 15%), we also found that the pulmonary vascular resistance was similar to ambient air breathing. In accordance with healthy volunteers and patients with cardiovascular diseases or COPD, exercise performance as assessed by the 6 MWD was reduced at altitude and irrespective of whether NOT or placebo were applied during the nights (11, 24).

We and others have demonstrated that SDB including nocturnal oxygen desaturation and periodic breathing are highly prevalent in patients with right heart failure due to PH of various etiologies and are associated with reduced quality of life, impaired exercise capacity, and hemodynamics (3, 34–36): 39% of 43 PH patients studied at our center had ≥ 10 events/h of Cheyne–Stokes respiration/central sleep apnea (CSR/CSA), and the majority of them (68%) spent more than 10% of the night time with a low arterial SpO_2_ (<90%). With the current study, we have shown that SDB in PH are even more pronounced at altitude. This is in line with healthy volunteers, patients with COPD, and highlanders permanently living above 2,600 m (29, 37, 38).

This trial included only stable PH patients in NYHA class II; therefore, this data may not apply to more severe PH patients, at higher altitudes or longer exposures. Patients which were included in this trial were relatively physically fit (median baseline 6 MWD 620 m at 490 m). How PH patients with worse exercise performance at low altitude would tolerate altitude with and without NOT remains to be studied. The sample size of 6 was too small to draw definitive conclusions on outcomes and to detect significant changes in pulmonary artery pressure and right heart function with altitude. However, this was an exploratory pilot study that paved the way to future larger trials.

## Conclusion

In conclusion, 7 of 9 stable PH patients were able to safely travel to 2,048 m. One patient suffered from syncope during maximal incremental exercise test and another from low oxygen saturation over a sustained period of time during the night needing oxygen therapy. Altitude exposure induced hypoxemia, impairment of exercise performance, and SDB. NOT improved SDB but had no sustained effect on exercise performance or echocardiographic parameters during daytime. Our pilot trial is an important basis for designing further trials evaluating altitude tolerance of PH patients.

## Data Availability Statement

The raw data supporting the conclusions of this article will be made available by the corresponding author upon request, without undue reservation.

## Ethics Statement

The studies involving human participants were reviewed and approved by Cantonal Ethics Committee Zurich (EK-2013-0088). The patients/participants provided their written informed consent to participate in this study.

## Author Contributions

ML, SS, and SU: analyzing and interpretation, drafting of the manuscript, and final approval of the version to be published. TL, SA, FH, PS, JH, SRS, EH, and MF: data acquisition, critical revision for important intellectual content, and final approval of the version to be published. ML, KB, and SU: conception and design of the study, data acquisition, interpretation of the data, critical revision for important intellectual content, and final approval of the version to be published. SU: guarantor of the paper. All authors contributed to the article and approved the submitted version.

## Conflict of Interest

The authors declare that the research was conducted in the absence of any commercial or financial relationships that could be construed as a potential conflict of interest.

## References

[1] Simonneau G Montani D Celermajer DS Denton CP Gatzoulis MA Krowka M. Haemodynamic definitions and updated clinical classification of pulmonary hypertension. Eur Respir J. (2018) 53:1801913. 10.1183/13993003.01913-2018 30545968 PMC6351336

[2] Ulrich S Fischler M Speich R Bloch KE. Wrist actigraphy predicts outcome in patients with pulmonary hypertension. Respiration. (2013) 86:45–51. 10.1159/000342351 23234873

[3] Ulrich S Fischler M Speich R Bloch KE. Sleep-related breathing disorders in patients with pulmonary hypertension. Chest. (2008) 133:1375–80. 10.1378/chest.07-3035 18339776

[4] Ulrich S Keusch S Hildenbrand FF Lo Cascio C Huber LC Tanner FC. Effect of nocturnal oxygen and acetazolamide on exercise performance in patients with pre-capillary pulmonary hypertension and sleep-disturbed breathing: randomized, double-blind, cross-over trial. Eur Heart J. (2015) 36:615–23. 10.1093/eurheartj/eht540 24366914

[5] Galie N Humbert M Vachiery JL Gibbs S Lang I Torbicki A 2015 ESC/ERS Guidelines for the diagnosis and treatment of pulmonary hypertension: The Joint Task Force for the Diagnosis and Treatment of Pulmonary Hypertension of the European Society of Cardiology (ESC) and the European Respiratory Society (ERS): Endorsed by: Association for European Paediatric and Congenital Cardiology (AEPC), International Society for Heart and Lung Transplantation (ISHLT). Eur Respir J. (2015) 37:67–119. 10.1183/13993003.01032-2015 26318161

[6] Ulrich S Schneider SR Bloch KE. Effect of hypoxia and hyperoxia on exercise performance in healthy individuals and in patients with pulmonary hypertension: a systematic review. J Appl Physiol. (2017) 123:1657–70. 10.1152/japplphysiol.00186.2017 28775065

[7] Seccombe LM Chow V Zhao W Lau EM Rogers PG Ng AC. Right heart function during simulated altitude in patients with pulmonary arterial hypertension. Open Heart. (2017) 4:e000532. 10.1136/openhrt-2016-000532 28123765 PMC5255554

[8] Groth A Saxer S Bader PR Lichtblau M Furian M Schneider SR. Acute hemodynamic changes by breathing hypoxic and hyperoxic gas mixtures in pulmonary arterial and chronic thromboembolic pulmonary hypertension. Int J Cardiol. (2018) 270:262–67. 10.1016/j.ijcard.2018.05.127 29891241

[9] Naeije R. Physiological adaptation of the cardiovascular system to high altitude. Prog Cardiovasc Dis. (2010) 52:456–66. 10.1016/j.pcad.2010.03.004 20417339

[10] Furian M Flueck D Latshang TD Scheiwiller PM Segitz SD Mueller-Mottet S. Exercise performance and symptoms in lowlanders with COPD ascending to moderate altitude: randomized trial. Int J Chron Obstruct Pulmon Dis. (2018) 13:3529–38. 10.2147/COPD.S173039 30464436 PMC6208550

[11] Furian M Hartmann SE Latshang TD Flueck D Murer C Scheiwiller PM. Exercise performance of lowlanders with COPD at 2,590 m: data from a randomized trial. Respiration. (2018) 95:422–32. 10.1159/000486450 29502125

[12] Latshang TD Turk AJ Hess T Schoch OD Bosch MM Barthelmes D. Acclimatization improves submaximal exercise economy at 5533 m. Scand J Med Sci Sports. (2013) 23:458–67. 10.1111/j.1600-0838.2011.01403.x 22093058

[13] Sampson JB Cymerman A Burse RL Maher JT Rock PB. Procedures for the measurement of acute mountain sickness. Aviat Space Environ Med. (1983) 54:1063–73. 6661120

[14] Ats ATS statement: guidelines for the six-minute walk test. Am J Respir Crit Care Med. (2002) 166:111–7. 10.1164/ajrccm.166.1.at1102 12091180

[15] Lichtblau M Latshang TD Furian M Muller-Mottet S Kuest S Tanner F. Right and left heart function in lowlanders with COPD at altitude: data from a randomized study. Respiration. (2019) 97:125–34. 10.1159/000492898 30269143

[16] Evangelista A Flachskampf F Lancellotti P Badano L Aguilar R Monaghan M. European Association of Echocardiography recommendations for standardization of performance, digital storage and reporting of echocardiographic studies. Eur J Echocardiogr. (2008) 9:438–48. 10.1093/ejechocard/jen174 18579482

[17] Latshang TD Nussbaumer-Ochsner Y Henn RM Ulrich S Lo Cascio CM Ledergerber B. Effect of acetazolamide and autoCPAP therapy on breathing disturbances among patients with obstructive sleep apnea syndrome who travel to altitude: a randomized controlled trial. JAMA. (2012) 308:2390–8. 10.1001/jama.2012.94847 23232895

[18] Schwarz EI Furian M Schlatzer C Stradling JR Kohler M Bloch KE. Nocturnal cerebral hypoxia in obstructive sleep apnoea: a randomised controlled trial. Eur Respir J. (2018) 51:1800032. 10.1183/13993003.00032-2018 29700104

[19] Sleep Disorders Atlas Task Force. EEG arousals: scoring rules and examples: a preliminary report from the Sleep Disorders Atlas Task Force of the American Sleep Disorders Association. Sleep. (1992) 15:173–84. 10.1093/sleep/15.2.174 11032543

[20] Nussbaumer-Ochsner Y Schuepfer N Ulrich S Bloch KE. Exacerbation of sleep apnoea by frequent central events in patients with the obstructive sleep apnoea syndrome at altitude: a randomised trial. Thorax. (2010) 65:429–35. 10.1136/thx.2009.125849 20435865

[21] Furian M Lichtblau M Aeschbacher SS Estebesova B Emilov B Sheraliev U. Effect of dexamethasone on nocturnal oxygenation in lowlanders with chronic obstructive pulmonary disease traveling to 3100 meters: a randomized clinical trial. JAMA Netw Open. (2019) 2:e190067. 10.1001/jamanetworkopen.2019.0067 30794302 PMC6484579

[22] Tan L Latshang TD Aeschbacher SS Huber F Flueck D Lichtblau M. Effect of nocturnal oxygen therapy on nocturnal hypoxemia and sleep apnea in patients with chronic obstructive pulmonary disease travelling to altitude. A randomized clinical trial. JAMA Netw Open. (2020) 3:e207940. 10.1001/jamanetworkopen.2020.7940 32568400 PMC7309443

[23] Dehnert C Bartsch P. Can patients with coronary heart disease go to high altitude? High Alt Med Biol. (2010) 11:183–8. 10.1089/ham.2010.1024 20919884

[24] Agostoni P. Considerations on safety and treatment of patients with chronic heart failure at high altitude. High Alt Med Biol. (2013) 14:96–100. 10.1089/ham.2012.1117 23795728

[25] Wyss CA Koepfli P Fretz G Seebauer M Schirlo C Kaufmann PA. Influence of altitude exposure on coronary flow reserve. Circulation. (2003) 108:1202–7. 10.1161/01.CIR.0000087432.63671.2E 12939217

[26] Schmid JP Nobel D Brugger N Novak J Palau P Trepp A. Short-term high altitude exposure at 3454 m is well tolerated in patients with stable heart failure. Eur J Heart Fail. (2015) 17:182–6. 10.1002/ejhf.227 25597947

[27] Schmid JP Noveanu M Gaillet R Hellige G Wahl A Saner H. Safety and exercise tolerance of acute high altitude exposure (3454 m) among patients with coronary artery disease. Heart. (2006) 92:921–5. 10.1136/hrt.2005.072520 16339809 PMC1860700

[28] De Vries ST Komdeur P Aalbersberg S Van Enst GC Breeman A Van 'T Hof AW. Effects of altitude on exercise level and heart rate in patients with coronary artery disease and healthy controls. Neth Heart J. (2010) 18:118–21. 10.1007/BF03091749 20390061 PMC2848353

[29] Latshang TD Tardent RPM Furian M Flueck D Segitz SD Mueller-Mottet S. Sleep and breathing disturbances in patients with chronic obstructive pulmonary disease traveling to altitude: a randomized trial. Sleep. (2019) 42:zsy203. 10.1093/sleep/zsy203 30517695

[30] Nussbaumer-Ochsner Y Latshang TD Ulrich S Kohler M Thurnheer R Bloch KE. Patients with obstructive sleep apnea syndrome benefit from acetazolamide during an altitude sojourn: a randomized, placebo-controlled, double-blind trial. Chest. (2012) 141:131–8. 10.1378/chest.11-0375 21659435

[31] Latshang TD Lo Cascio CM Stowhas AC Grimm M Stadelmann K Tesler N. Are nocturnal breathing, sleep, and cognitive performance impaired at moderate altitude. (1,630-2,590 m)? Sleep. (2013) 36:1969–76. 10.5665/sleep.3242 24293773 PMC3825448

[32] Ahmedzai S Balfour-Lynn IM Bewick T Buchdahl R Coker RK Cummin AR. Managing passengers with stable respiratory disease planning air travel: British Thoracic Society recommendations. Thorax. (2011) 66 (Suppl 1.):i1–30. 10.1136/thoraxjnl-2011-200295 21856702

[33] Howard LS. Last call for the flight simulation test? Eur Respir J. (2013) 42:1175–7. 10.1183/09031936.00037813 24178931

[34] Rafanan AL Golish JA Dinner DS Hague LK Arroliga AC. Nocturnal hypoxemia is common in primary pulmonary hypertension. Chest. (2001) 120:894–9. 10.1378/chest.120.3.894 11555526

[35] Schulz R Baseler G Ghofrani HA Grimminger F Olschewski H Seeger W. Nocturnal periodic breathing in primary pulmonary hypertension. Eur Respir J. (2002) 19:658–63. 10.1183/09031936.02.00225102 11998995

[36] Hildenbrand FF Bloch KE Speich R Ulrich S. Daytime measurements underestimate nocturnal oxygen desaturations in pulmonary arterial and chronic thromboembolic pulmonary hypertension. Respiration. (2012) 84:477–84. 10.1159/000341182 23011320

[37] Bloch KE Buenzli JC Latshang TD Ulrich S. Sleep at high altitude: guesses and facts. J Appl Physiol. (2015) 119:1466–80. 10.1152/japplphysiol.00448.2015 26229000

[38] Latshang TD Furian M Aeschbacher SS Ulrich S Osmonov B Mirrakhimov EM. Association between sleep apnoea and pulmonary hypertension in Kyrgyz highlanders. Eur Respir J. (2017) 49:1601530. 10.1183/13993003.01530-2016 28007792

